# Combination therapy of adagrasib and abemaciclib in non-small cell lung cancer brain metastasis models genomically characterized by KRAS-G12C and homozygous loss of CDKN2A

**DOI:** 10.1186/s40478-025-01993-2

**Published:** 2025-05-02

**Authors:** Christian Migliarese, Yinon Sadeh, Consuelo Torrini, Fatma Turna Demir, Naema Nayyar, Erika Yamazawa, Yuu Ishikawa, Nazanin Ijad, Elizabeth J. Summers, Adam Elliott, Lisa Rahbaek, Barbara Saechao, Jill Hallin, Priscilla K. Brastianos, Hiroaki Wakimoto

**Affiliations:** 1 Department of Neurosurgery, Massachusetts General Hospital, Boston, MA USA; 2 Cancer Center, Massachusetts General Hospital, Harvard Medical School, Boston, MA USA; 3 Medical Laboratory Techniques Programme, Department of Medical Services and Techniques, Vocational School of Health Services, Antalya Bilim University, Antalya, Turkey; 4 Mirati Therapeutics, Inc., a Bristol Myers Squibb Company, San Diego, CA USA

**Keywords:** Brain metastasis, Non-small cell lung cancer, KRAS-G12C mutation, CDKN2A/B homozygous deletion, Adagrasib, Abemaciclib, Combination therapy

## Abstract

KRAS mutations are prevalent in brain metastases (BM) from non-small cell lung cancer (NSCLC). The activity of KRAS-G12C selective, brain-penetrant small molecule inhibitor adagrasib was recently demonstrated in preclinical models of BM and patients with BM carrying KRAS-G12C, leading to a clinical trial investigating this therapeutic approach. However, co-existing genomic drivers such as homozygous deletion of *CDKN2A/B* may impact the utility of adagrasib. We therefore explored the combination therapy employing adagrasib and abemaciclib, a brain-penetrant CDK4/6 inhibitor, in NSCLC BM models driven by KRAS-G12C and *CDKN2A* loss. In both adagrasib-resistant SW1573 cells and adagrasib-responsive H2122 cells, combination of adagrasib and abemaciclib was slightly synergistic in inhibiting cell viability in vitro through targeting the KRAS-ERK and CDK4/6-Rb signaling pathways. Combination treatment was necessary to activate caspase 3/7-mediated apoptosis in SW1573 cells, while adagrasib alone and in combination comparably elicited apoptosis in H2122 cells. In vivo, combination treatment with adagrasib (75 mg/kg) twice daily and abemaciclib (50 mg/kg) daily was associated with body weight loss (about 10%) in mice bearing orthotopic BM derived with SW1573 or H2122 cells, requiring 50% dose reduction of adagrasib in some animals. Notably, combination treatment, but neither monotherapy, extended animal survival in the SW1573 model. On the other hand, adagrasib monotherapy and combination were similarly effective at prolonging survival, while abemaciclib monotherapy was ineffective in the H2122 model. Pharmacokinetic studies confirmed brain-penetrant properties of both agents and revealed drug-drug interactions as abemaciclib exposures in the plasma and brains were increased by the presence of adagrasib. Immunohistochemistry demonstrated on-target pharmacodynamic effects of both agents in BM in mice. Our work thus supports that the combination treatment of adagrasib and abemaciclib can offer a therapeutic strategy in NSCLC BM genomically characterized by KRAS-G12C and CDKN2A loss.

**Supplementary Information:**

The online version contains supplementary material available at 10.1186/s40478-025-01993-2.

## Introduction

Brain metastasis (BM) is a clinical challenge manifesting in approximately 20–40% of patients originating from diverse solid malignancies, notably lung (10–50%), melanoma (7–16%), and breast (5–20%) cancers [2, 3]. Non-small cell lung cancer (NSCLC) constitutes nearly half of all BM cases [4], imparting an unfavorable prognosis and emerging as the predominant cause of mortality in over 70% of NSCLC cases [5]. Greater than 40% of NSCLC patients develop BM over the course of the disease [6], and untreated patients have a median survival of just 2 months [7]. Traditionally, treatment options for individuals with BM encompass surgical resection, radiation, and chemotherapy [7–9]. However, efficacy is limited as the median overall survival spans from 4 to 9 months with treatment [8, 10–12].

Advances in identifying mutated driver oncogenes, such as EGFR and ALK, have opened up opportunities to develop selectively targeted molecular therapeutics to improve NSCLC patient outcomes. A member of the Rat sarcoma virus (Ras) gene family, KRAS is one of the most frequently mutated oncogenes, accounting for 31% of lung cancers [13], including over 40% of NSCLC cases. The KRAS gene product acts as a binary on–off switch between GDP/GTP to regulate various signaling networks, most notably MAPK signaling, and cellular processes such as proliferation, and its mutations drive oncogenesis [14]. Mutant KRAS significantly contributes to metastatic dissemination, with a prevalence of BM detected in 27%–42% of patients harboring KRAS-G12C [6, 15]. This has prompted a heightened interest in therapeutically intervening mutant KRAS proteins [16].

Adagrasib (MRTX849) is a potent and covalent KRAS-G12C inhibitor that exhibits selective modification of the mutated cysteine 12 residue of KRAS to lock the protein in its inactive GDP-bound state [1]. Favorable pharmacokinetic properties, including long half-life (24 h) and extensive tissue distribution, as well as ability to mediate tumor regression in preclinical models at clinically relevant concentrations [1] have led to its FDA approval. Furthermore, we have shown significant intracranial activity of adagrasib in BM mouse models of KRAS-G12C NSCLC, and preliminary but encouraging anti-tumor effects with effective cerebrospinal fluid (CSF) penetration in patients with KRAS-G12C BM [6]. These preclinical and clinical results have provided a strong rationale to investigate applications of adagrasib to patients with BM and allowed its inclusion in a genomically guided multicenter clinical trial (NCT03994796).

Despite the promise, however, resistance to adagrasib monotherapy has been reported in cancers harboring KRAS-G12C. Clinically, (1) secondary mutations or amplifications in *KRAS*, (2) alternative oncogenic alterations that activate the receptor tyrosine kinase–RAS signaling pathway, and (3) histologic transformation to squamous-cell carcinoma, have been reported to be putative mechanisms underlying acquired resistance [17]. From our previous study, we have identified the prevalence of *CDKN2A/B* homozygous deletion as a common driver in lung adenocarcinoma BM [18]. *CDKN2A* loss leads to hyperactivation of CDK4/6 signaling, inducing abnormal activity in the G1 to S phase cell cycle transition and ultimately promoting proliferation [19]. *CDKN2A* homozygous deletion provides an additional actionable target of interest [1], which is supported by the observation that its coexistence with KRAS-G12C is associated with early disease progression and poor clinical outcomes with KRAS-G12C inhibitors [20]. Availability of small molecule inhibitors of CDK4/6 enables a combination strategy that co-targets CDK4/6 and KRAS-G12C, and its potential was preclinically demonstrated in flank tumor models [1]. CDK4/6 inhibitor palbociclib demonstrated intracranial activity in patients with progressive BM with CDK pathway alterations [21]. Another potent and selective inhibitor of CDK4 and CDK6, abemaciclib, was recently approved by the FDA for treating hormone-receptor-positive breast cancer [22], and has shown the unique ability to penetrate the blood–brain barrier (BBB), achieving comparable concentrations in the CSF and plasma, clinically [23].

Here, the goal of our study is to address our hypothesis that combination therapy employing two brain penetrant agents adagrasib and abemaciclib will have intracranial efficacy in preclinical xenograft models of NSCLC BM genetically defined by the co-occurrence of KRAS-G12C and *CDKN2A* homozygous deletion. Specifically, we investigate the combination strategy in two human NSCLC-derived models that are either resistant or semi-sensitive to adagrasib monotherapy.

## Materials and methods

### Cell lines and cell cultures

Human NSCLC cell lines, SW1573 and H2122 cells, were obtained from the American Type Culture Collection. Both cell lines carry a KRAS G12C mutation and CDKN2A homozygous deletion. The cell lines were authenticated by short tandem fingerprinting in 2022 and periodically tested to be Mycoplasma-free. H2122 cells were cultured in RPMI-1640 supplemented with 10% fetal bovine serum. SW1573 cells were maintained in DMEM supplemented with 5% FBS. Both cell lines were cultured at 37 °C in a 5% CO_2_-humidified atmosphere and passaged when they reached 80–90% confluence. Cells were transduced with a lentivirus vector carrying cDNA coding for firefly luciferase and mCherry to generate SW1573-FmC and H2122-FmC for in vivo studies.

### Cell proliferation and viability assays (CyQUANT & Cell Titer Glo)

Cells were cultured in a 96-well plate at a density between 1,000 and 3,500 cells/well and allotted 24 h for adhesion to the well, which was followed by treatment with serially diluted adagrasib, abemaciclib, or both drugs in combination for 72 h (adagrasib), 120 h (abemaciclib), or 96 h (combination). Cell viability was measured using Cell Titer Glo for adagrasib and combination treatments, while Abemaciclib data were obtained using CyQUANT assay (ThermoFisher). Combination data was analyzed using Combenefit software employing the Highest Single Agent model [24].

### Western blot analysis

Cells and tissues were lysed in RIPA lysis buffer (Pierce). The protein concentration was measured using Bradford assay (BioRad). Protein lysates were separated by Mini-PROTEAN TGX gel electrophoresis (BioRad) and subsequently transferred to PVDF membranes (BioRad) for immunoblotting analysis. The membrane was blocked with 5% non-fat skim milk in Tris-buffered saline with tween. Incubation in diluted primary antibodies was conducted overnight and diluted horseradish peroxidase-conjugated secondary antibodies were subsequently applied. Immunoreactive bands were detected using ECL substrate reagent (BioRad), and the membrane was developed using a ChemiDoc XRS + system (BioRad). Primary antibodies used were against: phospho-ERK (Cell Signaling Technology (CST), 4370), ERK (CST, 9102), phospho-Rb (Ser807/811, CST, 8516), β-actin (CST, 3700), and vinculin (Millipore Sigma, 05–386).

### Cell cycle analysis

Cells were treated for 48 or 24 h with adagrasib, abemaciclib, or both drugs in combination. Cells were harvested, washed with PBS, and fixed in 2% paraformaldehyde. After the samples were prepared for analysis, fixed cells were resuspended in media and incubated with Vybrant DyeCycle Violet stain (ThermoFisher) for 30 min at 37 °C while protected from light. Flow cytometry was conducted using LSR II Cytometer (BD Biosciences) and analysis was done with FlowJo software and the Dean Jett-Fox algorithm.

### Caspase 3/7-Glo cell death assay

Cells were cultured in a white-walled 96-well plate at a density between 2,500 and 5,000 cells/well and allotted 24 h for adhesion to the well, which was followed by treatment with diluted adagrasib, abemaciclib, or both drugs in combination for 48 h. SW1573 received a concentration of 1,000 nM of adagrasib, 1,000 nM of abemaciclib, and 1,000 nM of both in combination. H2122 received a concentration of 50 nM or 1–100 nM of adagrasib, 600 nM of abemaciclib, and the same concentration in combination. Upon treatment of the cultured cells, 50 μL of the caspase3/7-Glo reagent was added per well, which was then mixed using a plate shaker and allowed to equalize to room temperature. Caspase activity was then measured using a plate reader to detect luminescence.

### Animal studies (xenograft models)

All the animal studies were conducted in compliance with all applicable regulations and guidelines of the Institutional Animal Care and Use Committee. Six-to-eight-week-old female nude (athymic) mice were obtained from Charles River Laboratory and maintained under pathogen-free conditions, with food and water provided ad libitum. Mice were implanted intracranially with SW1573-FmC (2 × 10^5^ in 2 μL serum-free media) or H2122-FmC cells (1.5 × 10^5^ in 2 μL serum-free media) in the right striatum region under general anesthesia using stereotactic procedures as previously described [6]. Animal health was monitored daily.

From three to four days after implantation, oral dosing of adagrasib (75 mg/kg) in vehicle (10% captisol in 50 mmol/L citrate buffer pH 5.0) twice daily (BID), abemaciclib (50 mg/kg) in 1% hydroxyethyl cellulose and 25 mM potassium phosphate monobasic buffer at pH 2.0 once daily (QD), or combination of both were performed. Treatments were given for 21 days plus an additional 14 days after a 6/9-day dose holiday. Animals were humanely euthanized when > 20% body weight loss or > 15% body weight loss plus neurological signs was noted. Survival data were collected for each group and analyzed using the Kaplan–Meier method. Survival duration by group was tested for statistical significance using the Gehan-Breslow-Wilcoxon test with False Discovery Rate correction. Statistical comparisons were considered significant when the adjusted P value was below 0.05 (*P* < 0.05).

For the pharmacokinetics/pharmacodynamic study, mice bearing SW1573-FmC or H2122-FmC intracranial tumors received oral doses of adagrasib (75 mg/kg) BID, abemaciclib (50 mg/kg) QD, or in combination, for three days. For SW1573-Fmc implanted mice, treatment began approximately 27 days post-implantation, with a total of 14 mice divided into vehicle (*n* = 3), adagrasib (*n* = 4), abemaciclib (*n* = 3), and combination treatment (*n* = 4). Treatment for H2122-Fmc implanted mice commenced approximately 30 days post-implantation, with a total of 13 mice divided into vehicle (*n* = 3), adagrasib (*n* = 3), abemaciclib (*n* = 3), and combination treatment (*n* = 4). Brains and whole blood were collected 1 h after the last dose for all groups. Plasma was separated from the blood collected in K2-EDTA tubes. Part of the contralateral hemisphere of the brain was excised, weighed, and acutely frozen. These samples were used for drug concentration analysis using Liquid Chromatography/Mass Spectrometry (LC/MS–MS). Paraffin blocks were prepared from the brains for H&E staining and immunohistochemistry.

### Bioluminescent imaging (BLI)

In vivo bioluminescent imaging (BLI) was performed 24 h prior to initial dosing with vehicle, adagrasib or abemaciclib, and additional imaging timepoints for all animals took place between 15 and 34 days after inoculation. Animals were injected intraperitoneally with luciferin (150 mg/kg, 0.01 mL/g) based on body weight and anesthetized using 1–2% isoflurane in oxygen at 1–2 L/minute. Images were acquired beginning at 7 min after luciferin injection for three time points (7, 15, and 30 min). Region of interest (ROI) analysis was completed on BLI images using AURA software (Spectral Instrument Imaging). BLI images were generated by overlaying BLI signals for each animal onto their respective white-light images for anatomic reference. Brain ROIs were generated using a fixed area circle and were placed based on the BLI signal in the relevant area, using the photographic anatomical reference images. BLI signals in images were scaled in units of radiance (photons per second per square millimeter per steradian). Prism 9 software (Graph Pad) was used to generate relevant plots based on the in vivo quantification.

### Bioanalysis of plasma, blood, and brain samples

Each brain sample and control were placed in a 2 mL round bottom Eppendorf tube and frozen. Brains were diluted tenfold with a 95/5 (water/ acetonitrile) solution, along with a 5 mm stainless steel bead then placed in a TissueLyser and shaken at 30 s/sec for 10 min. For standards and QCs, a 1 mg/mL DMSO solution was made of abemaciclib and adagrasib each in amber vials (protected from light). Each compound was further diluted in 50/50 (acetonitrile/water) to concentrations of 10–200,000 ng/mL. 10 μL of each concentration was then diluted with 90 μL of control mouse blood, plasma, or homogenized brain, for final concentrations of 1–20,000 ng/mL in each matrix. In a 2 mL 96 well plate, 10 μL of each sample, standard, QC, and control was pipetted into individual wells. 200 μL of acetonitrile (spiked with 100 ng/mL of each Labetalol and Verapamil as internal standards) was added in each well, shaken for 5 min, then centrifuged at 4,000 rpm for 5 min. 150 μL of the supernatant was transferred into a new plate with 150 μL of water added and shaken for another 5 min. Plates were loaded onto a LC–MS/MS system (Shimadzu, AB SCIEX) and analyzed and quantified using the Analyst software.

### Immunohistochemistry (IHC)

Animals harboring SW173-FmC intracranial tumors were treated with adagrasib (75 mg/kg, BID) and abemaciclib (50 mg/kg, QD) for three days and euthanized after 3 h following the last dose for brain harvesting. Formalin-fixed paraffin-embedded Sects. (7-μm thickness) were subjected to immunohistochemistry according to the methods described previously [6]. Primary antibodies used included those directed at: p-ERK (CST), p-Rb (CST), Ki67 (Dako, MIB1) and Cleaved caspase 3 (CST, 9661). Immuno-positive cells and negative cells within tumor tissues were counted and the fraction of positive cells determined.

### Statistical analyses

For the caspase 3/7 assay, differences in luminescence signals (from control) were determined using One-Way ANOVA. Differences in BLI radiance were tested for statistical significance using the One-Way ANOVA comparison. Statistical comparisons between two groups were considered significant when the adjusted P value was below 0.05 (*P* < 0.05). Kaplan–Meier analysis and log-rank test were used for the analysis of animal overall survival, comparing the groups.

## Results

### Adagrasib and abemaciclib combination demonstrates synergistic cytotoxicity in KRAS- G12C/CDKN2A mutant NSCLC cell lines

**Fig. 1:**
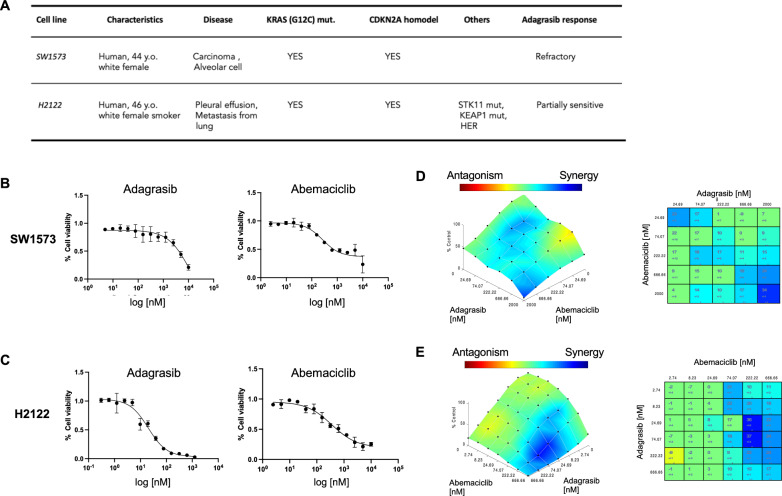
In vitro cell viability assays testing adagrasib and abemaciclib combinatorial treatment. **A**, Table representing the characteristics of the two cell lines [1]. **B**, **C**, CellTiter-Glo and CyQUANT assays to evaluate cell viability and proliferation, respectively, with adagrasib (72 h drug exposure) and abemaciclib (120 h drug exposure) monotherapies in SW1573 cells **B** and H2122 cells **C**. SW1573 cells, adagrasib IC50: 4,027 nM and abemaciclib IC50: 833 nM. H2122 cells, adagrasib IC50: 21.2 nM and abemaciclib IC50: 626 nM. **D, E,** Combenefit software analysis testing synergism of combining adagrasib and abemaciclib using Highest Single Agent model analysis in SW1573 **D** and H2122 **E** cell lines (96 h drug exposure)

To assess the antitumor efficacy of KRAS-G12C inhibitor (KRASi) adagrasib and CDK4/6 inhibitor (CDK4/6i) abemaciclib, individually and in combination, we used two NSCLC cell lines SW1573 and H2122 that both carry KRAS-G12C and *CDKN2A* homozygous deletion (Fig. 1A). These cell models were chosen because of their reported differential sensitivity to adagrasib that SW1573 is refractory while H2122 is partially resistant [1] (Fig. 1A). Monotherapy with adagrasib or abemaciclib was examined at escalating concentrations of each agent, using an adagrasib dose range covering higher concentrations (up to 10 µM) for SW1573, considering its resistant phenotype. Cell viability assay following 72-h drug exposure showed the 50% Inhibitory Concentration (IC50) of adagrasib greater than 4 µM in SW1573 cells (Fig. 1B), indicating the inefficacy of adagrasib monotreatment alone, consistent with a prior report [1]. In contrast, H2122 cells responded better to adagrasib, with an IC50 lower than 30 nM (Fig. 1C). Cell proliferation assay following 120-h abemaciclib monotreatment yielded an IC50 of 1 µM and 600 nM for SW1573 cells and H2122 cells, respectively (Fig. 1B, C), demonstrating a comparable response in the two cell lines.

To elucidate whether the combination of adagrasib and abemaciclib mediates a synergistic, additive, or antagonistic effect, we utilized Combenefit analysis. Combination treatment demonstrated an additive to synergistic effect in both SW1573 and H2122 cell lines (Fig. 1D, E), despite their differential sensitivity to adagrasib. These results highlight the beneficial anti-tumor effects resulting from the simultaneous inhibition of the two driver pathways, KRAS and CDK4/6, in these NSCLC cell lines. To confirm that lentiviral transduction with the firefly-luciferase mCherry (LV-FmC) did not alter drug responses, the same assays and analysis were conducted on the engineered cell lines, yielding results consistent with those obtained with the parental cells (Supplementary Fig. S1A-E).

### On-target signaling inhibition and cell death induction of combination treatment with adagrasib and abemaciclib in vitro

**Fig. 2:**
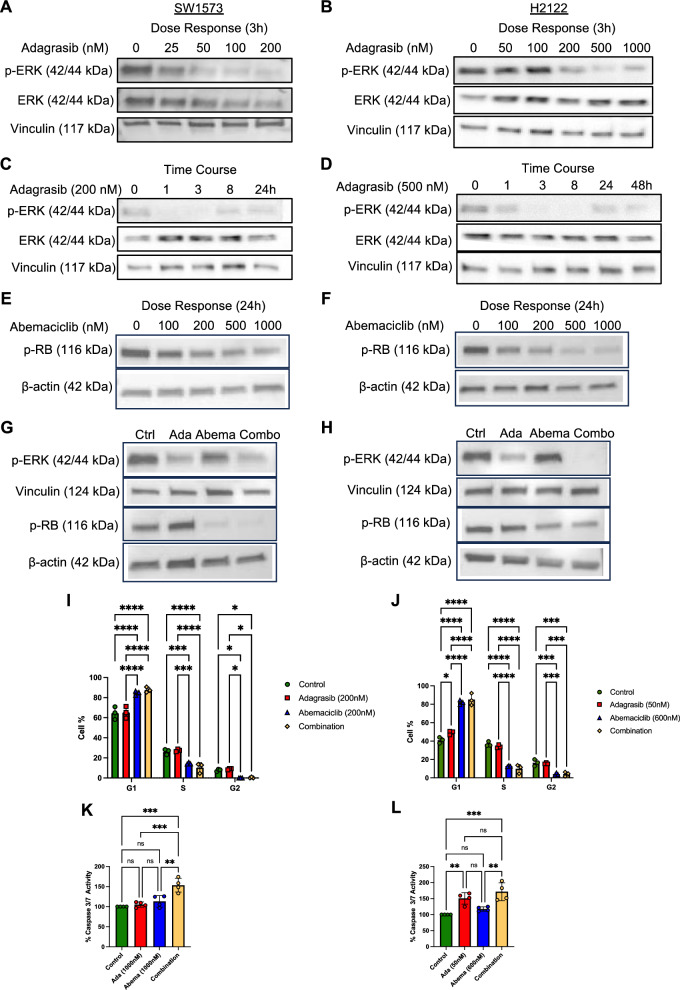
On target inhibition and apoptotic cell death induction by adagrasib and abemaciclib combinatorial treatment. **A, B,** Immunoblot analysis showing dose dependent effects of adagrasib (3 h exposure) on p-ERK in SW1573 cells **A** and H2122 cells **B**. **C, D,** Immunoblot analysis showing time dependent effects of indicated doses of adagrasib on p-ERK in SW1573 cells **C** and H2122 cells **D**. **E, F,** Immunoblot analysis showing dose dependent effects of abemaciclib (24 h exposure) on p-Rb in SW1573 cells **E** and H2122 cells **F**. **G,** Immunoblot analysis of SW1573 cells treated for 48 h with control, 1,000 nM adagrasib, 1,000 nM abemaciclib, and combination therapy. **H,** Immunoblot analysis of H2122 cells treated for 48 h with control, 100 nM adagrasib, 600 nM abemaciclib, and combination therapy. **I, J,** Cell cycle analysis in SW1573 and H2122 cells performed with the 4 groups: Control, Adagrasib, Abemaciclib and combination, for 24 h drug exposure at the indicated concentrations. Dean-Jett-Fox model analysis was used for G1, S, G2 cell cycle phase identification. Statistical analysis was with 2-way ANOVA multiple comparison between different groups phases using cell counts (%) after treatments: ^*^, Padj < 0.05;^**^, Padj < 0.005;^***^, Padj < 0.0005; ^****^, Padj < 0.00005. **K,** Caspase 3/7 activity in SW1573 cells treated for 48 h with control, 1,000 nM adagrasib (Ada), 1,000 nM abemaciclib (Abema), and combination therapy. **L,** Caspase 3/7activity in H2122 cells treated for 48 h with control, 50 nM adagrasib (Ada), 600 nM abemaciclib (Abema), and combination therapy

We used western blot analysis to characterize the inhibitory effects of each agent on target signaling pathways. Three-hour exposure to adagrasib inhibited phospho-ERK (p-ERK), downstream of KRAS in the MAPK signaling pathway, in both SW1573 cells (Fig. 2A) and H2122 cells (Fig. 2B) in a dose dependent manner. However, lower concentrations (as low as 25 nM) were needed to suppress p-ERK in SW1573 cells, despite their resistance to adagrasib monotherapy (Fig. 1B). Time course analysis of p-ERK showed strong inhibition for 1–3 h in both cells (Fig. 2C, D). Signaling reactivation emerged at 8 h in SW1573 cells (Fig. 2C) and at 24 h in H2122 cells (Fig. 2D), revealing a shorter duration of adagrasib effects in SW1573 cells. Twenty-four-hour-exposure to abemaciclib mediated dose-dependent inhibition of p-Rb (at Ser807 and Ser811), key molecular targets of CDK4/6, in SW1573 cells (Fig. 2E) and H2122 cells (Fig. 2F), exhibiting more potent inhibition in H2122 cells. Adagrasib inhibition of p-ERK and abemaciclib inhibition of p-Rb were retained when the cells were co-treated with the two agents (Fig. 2G, H). Interestingly, we observed that abemaciclib modestly suppressed p-ERK in SW1573 cells (Fig. 2G). Furthermore, suppression of p-ERK in H2122 cells was more potent with the combination therapy compared with adagrasib alone (Fig. 2H), suggesting a contribution of CDK4/6 signaling to p-ERK.

We next performed cell cycle analysis since CDK4/6 drives cell cycle progression by primarily accelerating the transition from the G1 to S phase of the cell cycle. Cells were exposed to adagrasib and/or abemaciclib for 24 h when they were subjected to flow cytometry-based cell cycle assays. Abemaciclib significantly increased the fraction of G1 phase and decreased the fractions of S and G2 phases in both SW1573 cells (Fig. 2I, Supplementary Fig. S2A) and H2122 cells (Fig. 2J, Supplementary Fig. S2B), the effects that were retained in the combination treatment (Fig. 2I, J). Adagrasib, on the other hand, had no effect on the cell cycle, except a small increase in the G1 phase observed in H2122 cells. Thus, abemaciclib inhibited the transition from the G1 to the S phase of the cell cycle in both SW1573 and H2122 cells, consistent with its on-target effect.

To examine whether adagrasib and/or abemaciclib induced caspase-dependent apoptosis, we used a caspase 3/7 activation assay following a 48-h drug exposure. In SW1573 cells, adagrasib and abemaciclib monotherapies did not significantly provoke activation of caspase 3/7 at 1,000 nM for each agent (Fig. 2K). However, the combination treatment significantly increased caspase 3/7 activity, compared to both control and monotherapy groups, indicative of apoptosis induction (Fig. 2K). On the other hand, the adagrasib-sensitive H2122 cells responded to both adagrasib monotherapy (at a much lower dose of 50 nM) and the combination treatment with increases in caspase 3/7 activity compared to the control (Fig. 2L). With the doses tested, adagrasib monotherapy and the combination treatment showed no statistically significant difference in caspase 3/7 activity.

These results underscore the differential responsiveness of the two cell lines to mono- and combination treatments.

### Combination therapy with adagrasib and abemaciclib demonstrates antitumor activity and prolonged survival in the KRAS G12C/CDKN2A mutant SW1573 xenograft model

**Fig. 3:**
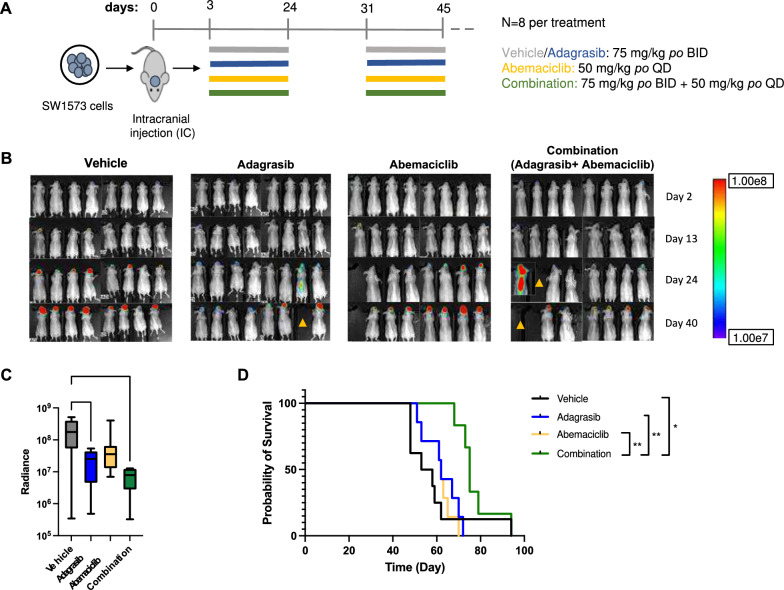
Combinatorial administration of adagrasib and abemaciclib increased animal survival in the KRASG12C/CDKN2A mutant SW1573 brain tumor model. **A**, Schematic representation of the study design. Treatment protocol involved administration of adagrasib at 75 mg/kg BID, abemaciclib at 50 mg/kg daily, or their combination via oral gavage to athymic mice with intracranially injected SW1573 cell line xenografts (*n* = 8/group). The treatment spanned for a total of 5 weeks. **B,** Bioluminescence Imaging signal detection using Aura software was performed at different time points (2, 13, 24, and 40 days) following intracranial injection. Yellow triangles indicate mice exhibiting spinal signals at pretreatment (See Supplementary Fig. 4B).** C**, Bioluminescence Imaging analysis at the 40-day time point. Statistical analysis revealed a significant decrease in signal for the combination and adagrasib groups compared to the vehicle group (^*^, Padj < 0.05). **D**, Survival data were collected for each group and analyzed by Kaplan–Meier statistical analysis. ^*^, Padj < 0.05; ^**^, Padj < 0.005. Animals that exhibited pre-treatment spinal disease were excluded from the analysis in **C** and **D**

We next investigated the anti-tumor efficacy of adagrasib and abemaciclib in intracranial tumor models carrying KRAS-G12C and CDKN2A loss. First, we employed the SW1573-FmC model, and randomized tumor-bearing animals into 4 groups: vehicle control, adagrasib (75 mg/kg, BID) alone, abemaciclib (50 mg/kg, QD) alone, and combination of the two agents. Treatments were given for a total of 5 weeks, with a one-week drug break between the first 3-week and the second 2-week courses. (Fig. 3A). During the treatment, we observed body weight loss (about 5–10%) in some of the mice in the combination group. According to our dosing criteria, animals that had > 8% body weight loss received diet gel and the half dose (morning alone) of adagrasib (Supplementary Fig. S3). This dose adjustment and the one-week interruption during treatment helped animals regain weight (Supplementary Fig. S3) and complete the total regimen of 5 weeks. Therefore, we considered the observed, reversible weight loss attributable to toxicities associated with the combination treatment regimen. Our initial analysis of overall survival indicated no statistically significant differences in the survival times between the groups (Supplementary Fig. S4A). We then re-assessed pre-treatment BLI in the whole body and identified three animals, one in the adagrasib alone and two in the combination groups, that had luciferase signals from the spinal cord (Supplementary Fig. S4B), revealing spinal dissemination likely caused by dispersed tumor cells at the time of intracranial implantation. All these three animals exhibited early and rapid health deterioration, did not respond to treatments, and died earlier than any of the vehicle-treated animals having no initial spinal disease (Fig. 3B, Supplementary Figs. S3 and S4). These observations led us to consider that pre-treatment tumor cell dissemination to the spinal cord made the disease model more aggressive than the model restricted to intracerebral disease and contributed a confounding factor to efficacy analysis. Thus, we excluded these mice with initial spinal disease and reanalyzed the BLI and survival data. Our BLI analysis showed a significant reduction in signal intensity (radiance) in the adagrasib and combination groups compared to the vehicle group on day 40 when the 5-week treatment was about to end, with a trend of the lowest signals in the combination group (Fig. 3B, C). The abemaciclib group had a lower average BLI compared to the vehicle, without statistical significance. Survival analysis demonstrated a significant extension of overall survival in the combination group compared with the vehicle and monotherapy groups (Fig. 3D). Monotherapy groups, however, did not show survival benefit over the vehicle group (Fig. 3D). Thus, in the adagrasib resistant SW1573 model harboring KRAS-G12C and *CDKN2A* homozygous loss, adagrasib and abemaciclib combination, but not adagrasib alone, mediated anti-tumor effects and survival prolongation.

### Adagrasib monotherapy and combination therapy with adagrasib and abemaciclib demonstrate comparable antitumor activity in the KRAS G12C/CDKN2A mutant H2122 xenograft model

**Fig. 4:**
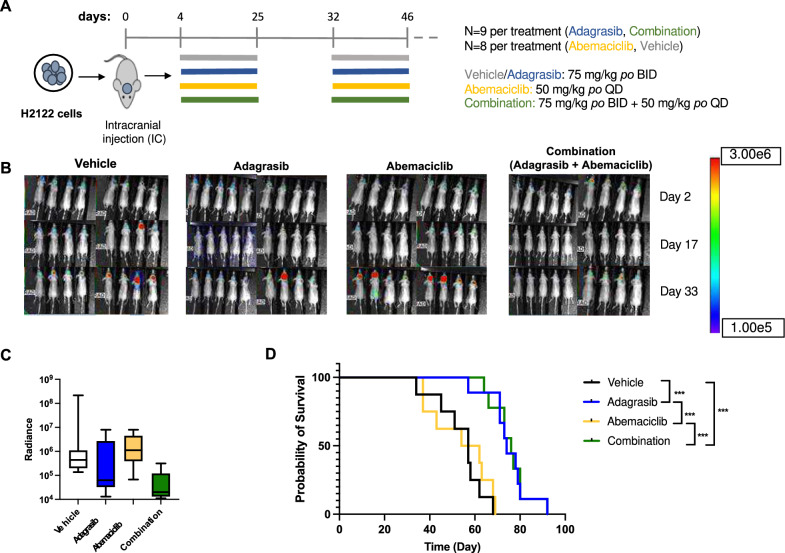
Adagrasib monotherapy and combination of adagrasib and abemaciclib increased animal survival in the KRASG12C/CDKN2A mutant H2122 brain tumor model. **A**, Schematic representation of the study design. The treatment protocol involved administering adagrasib at 75 mg/kg BID, abemaciclib at 50 mg/kg daily, or their combination via oral gavage to athymic mice with intracranially injected H2122 cell line xenografts (*n* = 8/group). Treatment spanned for a total of 5 weeks. **B,** Bioluminescence Imaging signal detection using Aura software was performed at different time points (2, 13, 24, and 40 days) following intracranial injection. **C**, Bioluminescence Imaging analysis was performed at day-40 time point. Comparisons between groups revealed no statistically significant differences. **D,** Kaplan–Meier survival analysis of all groups demonstrated that the combination and adagrasib treatment led to a statistically significant increase in survival compared with the abemaciclib alone and the vehicle groups. ^*^, Padj < 0.05; ^**^, Padj < 0.005; and.^***^, Padj < 0.0005

We next assessed the efficacy of combination therapy of adagrasib and abemaciclib employing a second orthotopic xenograft model with H2122-FmC cells. The same treatment regimens as above, involving adagrasib (75 mg/kg, BID) and abemaciclib (50 mg/kg, QD) either alone or combination were tested over a total of 5-week period (3 weeks and 2 weeks) (Fig. 4A). One animal of the adagrasib alone group and most of the combination group lost body weight (about 10%) during the second treatment cycle and were given diet gel and half adagrasib dose (morning only) to manage the apparent drug-related toxicities (Supplementary Fig. S5). Comparative BLI analysis of tumor burden revealed an apparent reduction of post-treatment signals in both adagrasib alone and combination groups, as compared to the control group, despite lack of statistical significance (Fig. 4B, C). The abemaciclib group on the other hand had signals comparable to the control (Fig. 4B, C). Kaplan–Meier survival analysis showed that adagrasib monotherapy and combination therapy similarly and significantly extended animal survival compared with the control group (Fig. 4D), in accord with the tendencies of tumor growth inhibition in the groups treated with adagrasib. In contrast, abemaciclib monotherapy provided no survival efficacy compared to the vehicle group (Fig. 4D). Therefore, in the H2122 tumor model that was responsive to adagrasib in vitro, adagrasib monotherapy and combination of adagrasib and abemaciclib were comparably effective at inhibiting tumor growth in vivo, and combining abemaciclib with adagrasib with the doses tested did not confer additional benefits.

### Brain-penetrant properties of adagrasib and abemaciclib and drug-drug interactions in mice

**Fig. 5:**
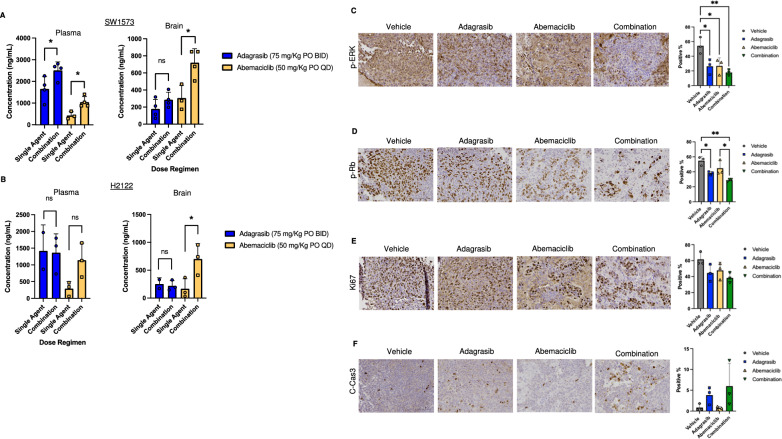
Pharmacokinetics and Pharmacodynamics of adagrasib and abemaciclib in xenograft brain metastasis mouse models. The treatment protocol involves administering adagrasib at 75 mg/kg BID, abemaciclib at 50 mg/kg daily, or their combination via oral gavage to n/n mice with intracranially injected H2122 (150,000 cells) or SW1573 (200,000 cells) cell line xenografts (*n* = 3/group, n = 4/combination group). The treatment spans a total of 3 days, and blood and brain collection were performed 3 h after the last dose. For the vehicle group, sample collection was made at 1 h after the last dose. Pharmacokinetic samples were collected from the plasma and right hemisphere of the brain, while pharmacodynamic samples were collected from the right hemisphere. **A**, Pharmacokinetics data plots illustrate drug concentration in plasma and brain samples of H2122 xenograft models, comparing single agents with combination treatment. **B,** Pharmacokinetics data plots illustrate drug concentration in plasma and brain samples of SW1573 xenograft models, comparing single agents with combination treatment. **C,** Pharmacodynamics, IHC, and quantitative data of cellular markers, p-ERK, p-Rb, Ki67, and C-Cas3 (Cleaved caspase 3), along with data plots illustrating the percentage of positive cells (%) between the groups in brain samples of the SW1573 xenograft-bearing mice used in the pharmacokinetic study

Next, we conducted pharmacokinetic (PK) studies in H2122-FmC and SW1573-FmC tumor-bearing mice. Plasma and brain samples were prepared 1 h after the last oral dosing of three-day treatment with adagrasib and abemaciclib. In both tumor models, adagrasib concentrations in plasma and brain were in the range of 1,400–2,500 and 180–300 ng/mL, respectively (Fig. 5AB), which was consistent with our prior report in a different tumor model (H23)^6^ and confirmed good brain exposure. Co-treatment with abemaciclib did not obviously impact adagrasib exposure but appeared to modestly increase both plasma and brain concentrations in the SW1573-FmC model. Plasma levels of abemaciclib were lower than adagrasib within the range of 290–400 ng/mL (Fig. 5AB). However, abemaciclib concentrations in the brain ranged from 200 to 300 ng/mL, demonstrating its ability to cross the blood–brain barrier [25, 26]. Interestingly, we observed notable elevations (about 2–4-fold) of abemaciclib exposure in the plasma and the brain in both tumor models when adagrasib was co-administered. Thus, our PK study validated the brain-penetrating capacity of adagrasib and abemaciclib and suggested drug-drug interactions when the two agents were administered simultaneously.

### Pharmacodynamic activities of adagrasib and abemaciclib in the SW1573 brain tumor model

Lastly, we explored pharmacodynamics in the SW1573-FmC xenograft model in which adagrasib combination with abemaciclib mediated survival efficacy indicative of combination benefit. Animals bearing orthotopic SW1573-FmC xenografts were treated with vehicles, adagrasib, abemaciclib or combination for 3 days before harvesting the brains for tissue sections. We conducted IHC for p-ERK, p-Rb, Ki67 and cleaved caspase 3 (C-Cas3) to examine treatment-induced molecular effects in the tumors. Quantitative analysis of p-ERK showed a decrease of p-ERK-positive cells in tumors treated with adagrasib and combination (Fig. 5C), consistent with downstream ERK inhibition by on-target adagrasib inhibition of KRAS-G12C. Interestingly, abemaciclib monotherapy also decreased p-ERK-positive cells (Fig. 5C), consistent with in vitro western blot results (Fig. 2. Abemaciclib monotherapy modestly decreased P-Rb immunopositivity despite the lack of statistical difference (Fig. 5D). Unexpectedly, adagrasib decreased p-Rb-positive cells, and the combination therapy was most potent to suppress p-Rb in the tumor (Fig. 5D). Frequency of cellular proliferation marker Ki67 mirrored the trends observed with P-Rb, with the combination group exhibiting the lowest Ki67 index (Fig. 5E). C-Cas3 exhibited a trend of increased positivity in the adagrasib alone and combination groups, reflecting induction of apoptosis, compared to the vehicle and abemaciclib alone groups both exhibiting very few C-Cas3 positive cells (Fig. 5F).

Collectively, these data showed on-target activity of adagrasib and abemaciclib in an NSCLC model in the brain. Overall, combination therapy demonstrated most effective anti-cancer properties over monotherapies, aligning with its survival benefit.

## Discussion

We previously reported that adagrasib demonstrated potent preclinical activity in multiple preclinical BM models of NSCLC harboring KRAS-G12C and preliminary clinical efficacy in patients with KRAS-G12C brain metastatic NSCLC [6], leading to inclusion in a genomically-guided clinical trial for patients with BM (NCT03994796, Alliance A071701). However, its efficacy against cancer BM having co-mutated genomic drivers has not been defined. Deletions in *CDKN2A/B* are one of the most frequent drivers in BM from NSCLC [18], making the resulting hyperactivation of the CDK4/6-Rb signaling an actionable target. Herein, we explored our hypothesis that the combination approach using selective and brain-penetrant inhibitors, adagrasib and abemaciclib, would be effective for BM models of NSCLC with the genomic alterations of KRAS-G12C and *CDKN2A* loss. Employing two NSCLC models of SW1573 and H2122 that show resistance and sensitivity to adagrasib monotherapy, respectively, we observed that the combination treatment was additive to slightly synergistic in both models in vitro. In vivo in intracranial tumor models, however, combination therapy was beneficial in the adagrasib-resistant SW1573 model, while combination therapy and adagrasib monotherapy were similarly effective in the adagrasib-sensitive H2122 model. Thus, despite similar genetic backgrounds, the two tumor models responded differently to adagrasib and the combinatorial strategy.

In vitro, adagrasib showed potent on-target inhibition of p-ERK in both models. Indeed, even more potent signaling inhibition was seen in the resistant SW1573 cells, revealing discordance with cell viability effects. However, rather rapid signaling reactivation was noted in SW1573 cells, which might underlie the resistant phenotype. Abemaciclib, on the other hand, showed comparable on-target inhibition in both models in vitro, based on its inhibition of p-Rb and G1 to S phase cell cycle transition. Interestingly, abemaciclib modestly suppressed p-ERK in SW1573 cells in vitro and in vivo, suggesting its intervention in the crosstalk between CDK4/6 and ERK signaling pathways. This finding is in contrast with compensatory upregulation of p-ERK induced by CDK4/6i reported in KRAS-mutant pancreatic cancer [27]. Evidence for signaling crosstalk was also found in H2122 cells, in which p-ERK was most potently reduced by combination therapy. This, however, did not translate into in vivo efficacy in this model.

In vivo, we noted that combination of adagrasib and abemaciclib with the doses used herein was associated with body weight loss (about 10%) of the treated animals that was likely attributable to drug-induced toxicities. We managed this issue by reducing the adagrasib dosing to half, which helped prevent further health deterioration and continue dosing of both agents. Despite the observed fluctuations in body weights, all mice completed the treatment protocol and fast recovery was noted after treatment cessation. Notably, the SW1573 and H2122 models responded differently to the treatments. In the adagrasib resistant SW1573 model, only the combination therapy was able to extend the survival, while both monotherapies were ineffective. A few animals having disseminated spinal disease at the time of treatment initiation responded poorly. On the other hand, in the adagrasib responsive H2122 model, adagrasib, but not abemaciclib, monotherapy increased animal survival and there was no additional survival benefit with abemaciclib in the combination-treated group. Although adagrasib monotherapy efficacy or lack thereof in vivo was as predicted from in vitro assays, various in vitro results such as synergy assays, western blot and cell cycle assays were not necessarily predictive of combination benefit in vivo. We noted, however, that caspase-dependent apoptosis activation in vitro was in accord with in vivo survival effects, identifying a potential biomarker that needs further research before drawing a conclusion. The lack of monotherapy efficacy with abemaciclib in both models aligns with prior reports suggesting that the optimal biomarkers for predicting response to CDK4/6 inhibitors are still not defined [22]. Cancer clinical trials of CDK4/6 inhibitors have shown conflicting evidence as some have shown association of CDKN2A homozygous loss with treatment response [21, 26, 28], but other have not [29–32]. Ongoing research efforts are focusing on understanding the role of tumor type, co-existing genomic alterations, and aberrant activation of CDK2 [33] as potential biomarkers of response and resistance to CDK4/6 inhibition.

Our pharmacokinetic and pharmacodynamic studies confirmed the ability of both agents to penetrate the brain and mediate on-target activity in tumors in the brain. The observed increase in abemaciclib concentrations, particularly in the brain, during co-administration with adagrasib can likely be attributed to their P-glycoprotein (p-gp) inhibitory effects [6, 34]. Both compounds are substrates and inhibitors of human p-gp-mediated efflux [6, 34], and this shared property likely contributed to the elevation of abemaciclib concentrations in the presence of adagrasib. Furthermore, considering adagrasib’s time-dependent inhibition of human CYP3A4, and abemaciclib being a substrate of CYP3A4 [34], our PK data suggested adagrasib-mediated inhibition of CYP3A4 in mice. This interaction adds another layer to the complexity of drug metabolism and requires understanding the interplay between different metabolic pathways when assessing combination therapies. Drug metabolism interactions can have multiple impacts. First, the enhanced drug penetration and exposure in the brain when administered simultaneously can contribute to the improved anti-tumor activity of the combination treatment that we found in the SW1573 model. Second, increased exposure may have contributed to the adverse events that were observed in the animals receiving adagrasib and abemaciclib concurrently, necessitating dose reduction. Thus, future research should focus on a better understanding of drug pharmacokinetics interactions to achieve alleviating treatment-associated toxicities and maximizing therapeutic impact.

We acknowledge that testing only two NSCLC models is one of the limitations in the current work. Since both models had CDKN2A homozygous loss, no firm conclusion can be made about the relationship of CDKN2A loss to CDK4/6 monotherapy and combination therapy response. Although we found activity of the combination therapy in the adagrasib resistant SW1573 model, this does not clarify that the patients with KRAS G12C mutant NSCLC brain metastasis that are resistant to KRAS G12C inhibitors are more likely to benefit from the combination therapy. In preclinical models of KRAS-mutant pancreatic ductal adenocarcinoma, co-inhibition of CDK4/6 and MAPK signaling pathway improved treatment efficacy [27]. Multiple clinical trials are underway to assess the potential of combining KRAS-G12C inhibitors with CDK4/6 inhibitors in solid cancers (NCT05178888 and NCT05358249) and expected to provide insight into biomarkers of response and resistance [35]. Given the frequent CDKN2A/B loss in brain metastases, this combinatorial approach could be beneficial for further investigation in patients with brain metastases to address a critical unmet clinical need in this challenging subset of patients.

## Conclusions

We show that the combination therapy of adagrasib and abemaciclib led to good brain penetration and on-target activity in brain tumors carrying *KRAS-G12C* and *CDKN2A* loss and was able to provide survival benefit in an NSCLC brain metastasis model that did not respond to monotherapy. Identifying CDK4/6 as a potentially actionable target in the context of KRAS-mutant NSCLC brain metastasis, our work provides a foundation for developing treatments for *KRAS-G12C/CDKN2A* mutant brain metastatic patients. Our work also underscores the importance of understanding patients’ genetic backgrounds and utilizing BBB-penetrant drugs. However, the ongoing challenges and opportunities in advancing targeted therapies for this patient population include understanding the drug-drug interactions, thorough safety assessments and dosing optimization, and identifying biomarkers predictive of therapeutic response.

## Supplementary Information

Supplementary material 1.

## Data Availability

Data is provided within the manuscript or supplementary information files.

## Abbreviations

BM: Brain metastasis
NSCLC: Non-small cell lung cancer
CSF: Cerebrospinal fluid
BBB: Blood-brain barrier
FmC: Firefly luciferase and mCherry
IC50: 50% Inhibitory concentration
BID: Twice daily
QD: Once daily
LC/MS-MS: Liquid chromatography/mass spectrometry
BLI: Bioluminescence imaging
ROI: Region of interest
CST: Cell signaling technologies
IHC: Immunohistochemistry
PK: Pharmacokinetic
p-gp: P-glycoprotein
