# Supplementary material for: Combination therapy of adagrasib and abemaciclib in non-small cell lung cancer brain metastasis models genomically characterized by KRAS-G12C and homozygous loss of CDKN2A
Source: Acta Neuropathol Commun. 2025 May 2;13:88. doi: 10.1186/s40478-025-01993-2 (PMC12046717; doi:10.1186/s40478-025-01993-2)
Supplement: Supplementary file 1 — Supplementary material 1. [file 40478_2025_1993_MOESM1_ESM.pdf]

Supplementary Material

Combination therapy of adagrasib and abemaciclib in non-small cell lung cancer brain metastasis models genomically characterized by KRAS-G12C and homozygous loss of CDKN2A

Christian Migliarese, Yinon Sadeh, Consuelo Torrini, Fatma Turna Demir, Naema Nayyar, Erika Yamazawa, Yuu Ishikawa, Nazanin Ijad, Elizabeth J. Summers, Adam Elliott, Lisa Rahbaek, Barbara Saechao, Jill Hallin, Priscilla K. Brastianos, and Hiroaki Wakimoto

Supplementary Figure 1

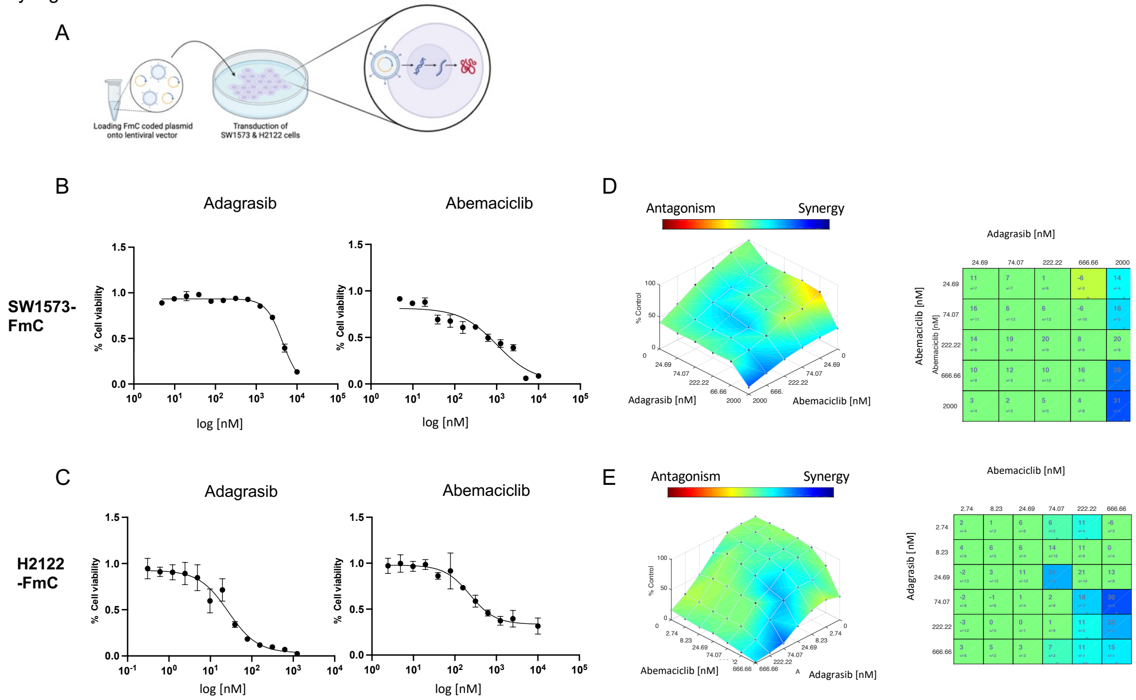

Supplementary Fig. 1. Combinatorial treatment with adagrasib and abemaciclib testing the cell viability of firefly-luciferase and mCherry (FmC)-transduced NSCLC cells.

A, Image representing cell line transduction procedures, involving overnight exposure with a lentivirus vector expressing Firefly luciferase and mCherry. **B, C,** CellTiter-Glo and CyQUANT assays to evaluate cell viability and proliferation, respectively, with adagrasib (72h drug exposure) and abemaciclib (120h drug exposure) monotherapies in SW1573-FmC cells (**B**) and H2122-FmC cells (**C**). SW1573-FmC cell line, adagrasib IC50: 4,379 nM and abemaciclib IC50: 1,000 nM. H2122-FmC cell line, adagrasib IC50: 28.54 nM and abemaciclib IC50: 518 nM. **D, E,** Combenefit software analysis testing synergism of combining adagrasib and amebaciclib using Highest Single Agent model analysis in SW1573-FmC (**D**) and H2122-FmC (**E**) cell lines (96h drug exposure).

Supplementary Fig. 2

A

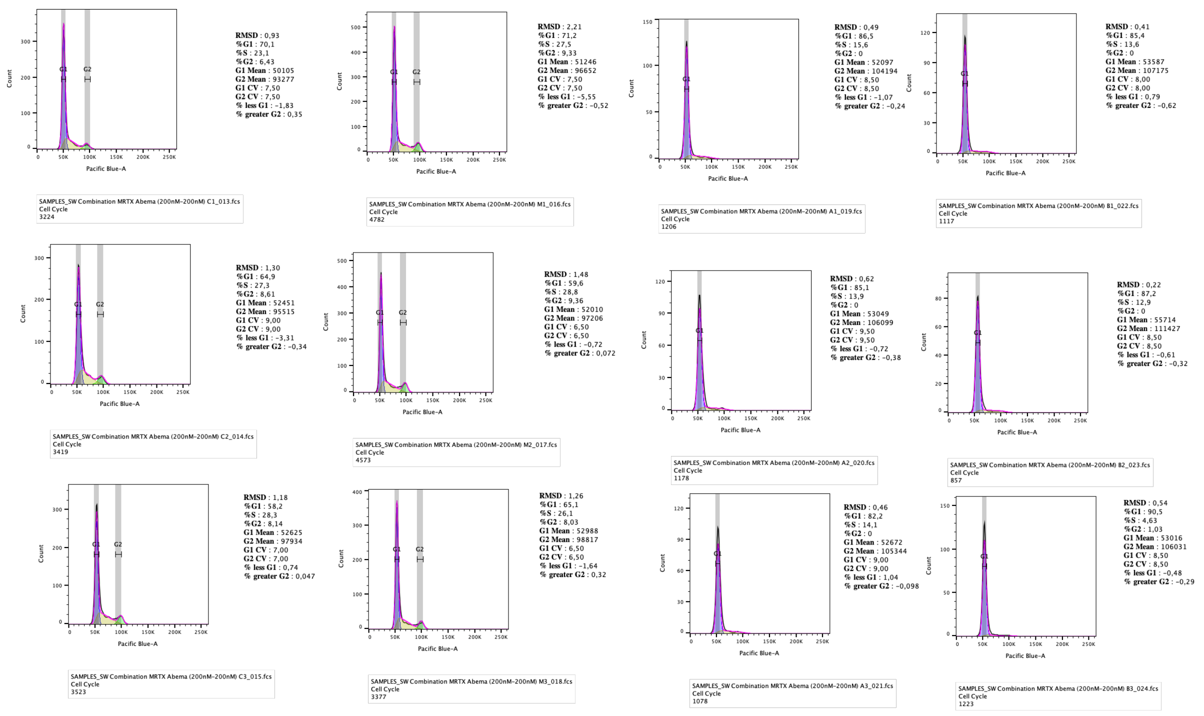

B

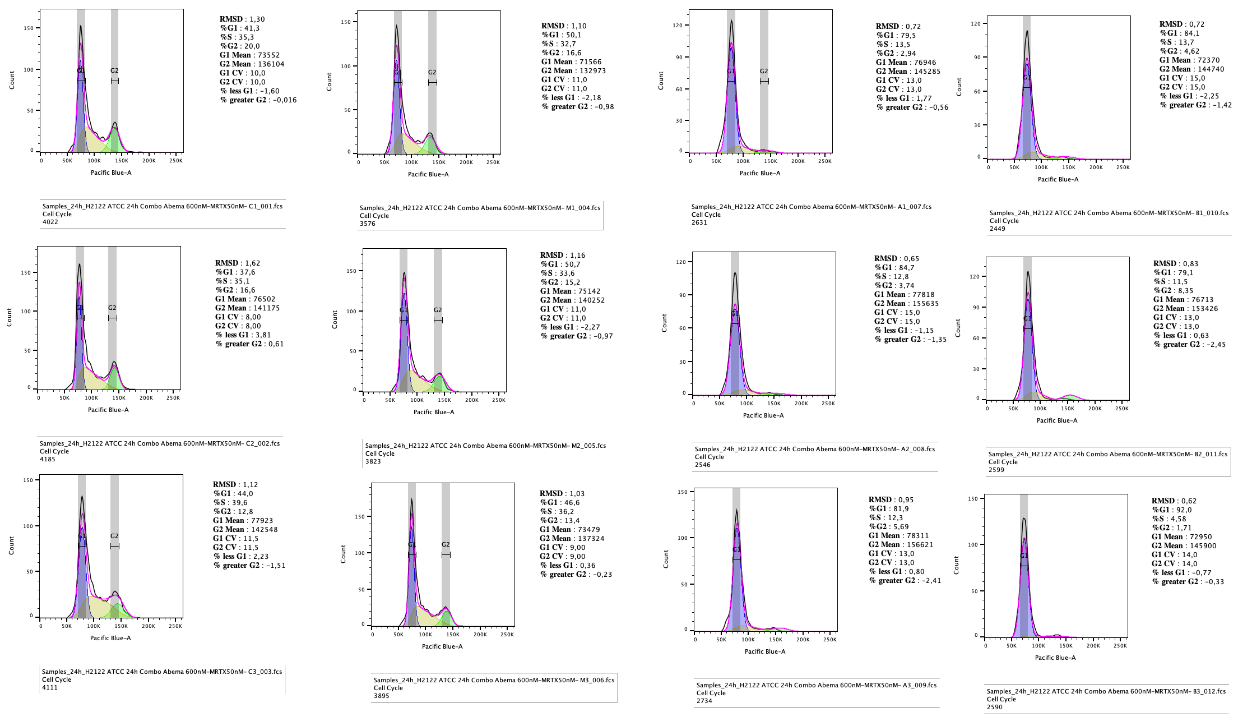

Supplementary Fig. 2. Cell cycle analysis of NSCLC cells treated with adagrasib, abemaciclib and combination. A, SW1573 cells. **B,** H2122 cells. See Figure 2I, J for quantitation of the data.

Supplementary Fig. 3

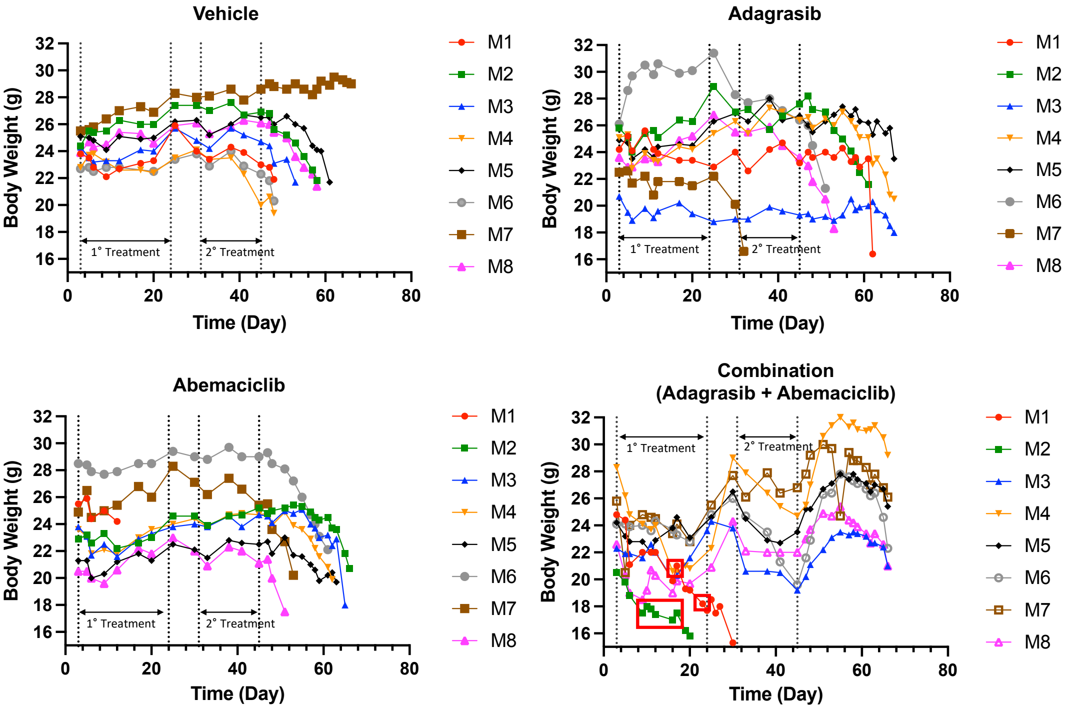

Supplementary Fig. 3. Body Weight Data (related with Figure 3). Mouse health monitoring with body weight measurements over the course of treatments. Mice were euthanized according to the criteria such as a 20% body weight loss, neurological signs, or a 15% body weight loss plus other signs. Treatment time periods, (first treatment from day 3 to day 24, second from day 31 to day 45), are represented by the dotted lines. Adagrasib evening dosing was skipped in mice with impaired health conditions for the time period (days) shown by the red boxes.

Supplementary Figure 4

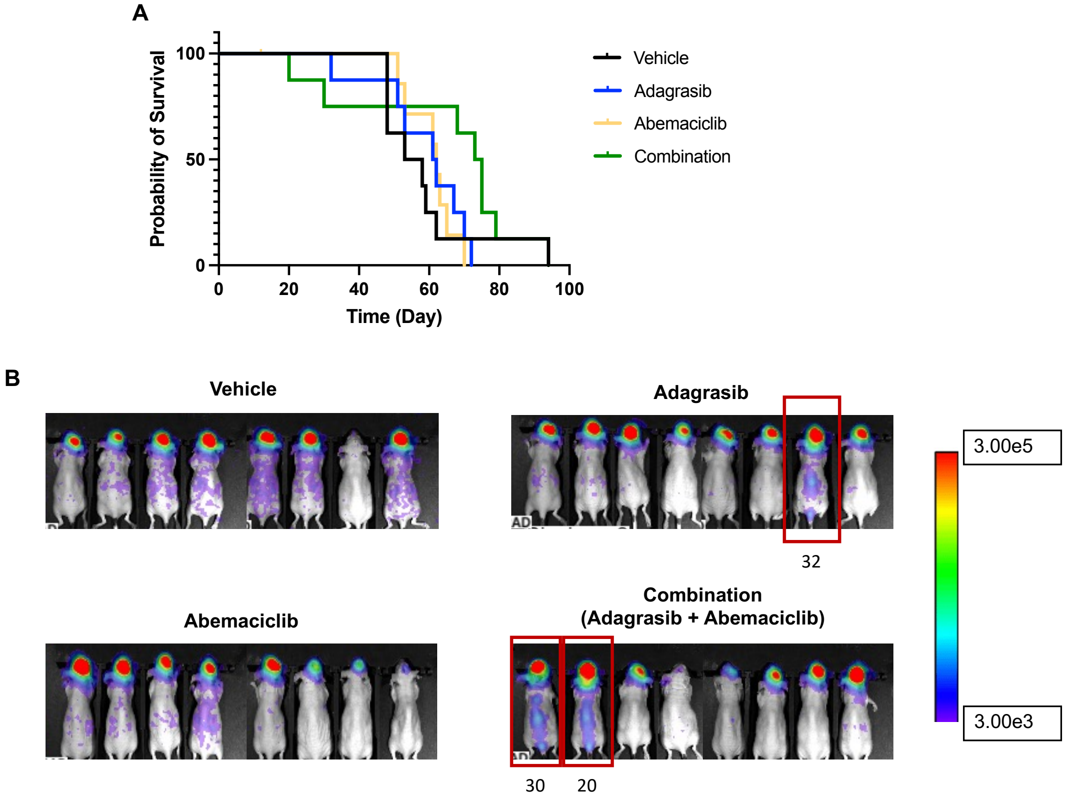

Supplementary Fig. 4. Combinatorial treatment with adagrasib and abemaciclib in the KRASG12C/CDKN2A mutant SW1573 brain tumor model (related with Figure 3). **A,** Kaplan-Meier survival analysis of all groups including all the animals. No statistical significance was noted in comparison of the groups. **B,** Bioluminescence Imaging signal detection using Aura software performed on Day 2 following intracranial injection before initiating treatments. Animals in the red box showed signals from the spinal cord, indicative of tumor cell dispersal at the time of implantation. Numbers: survival time (days) of these animals.

Supplementary Fig. 5

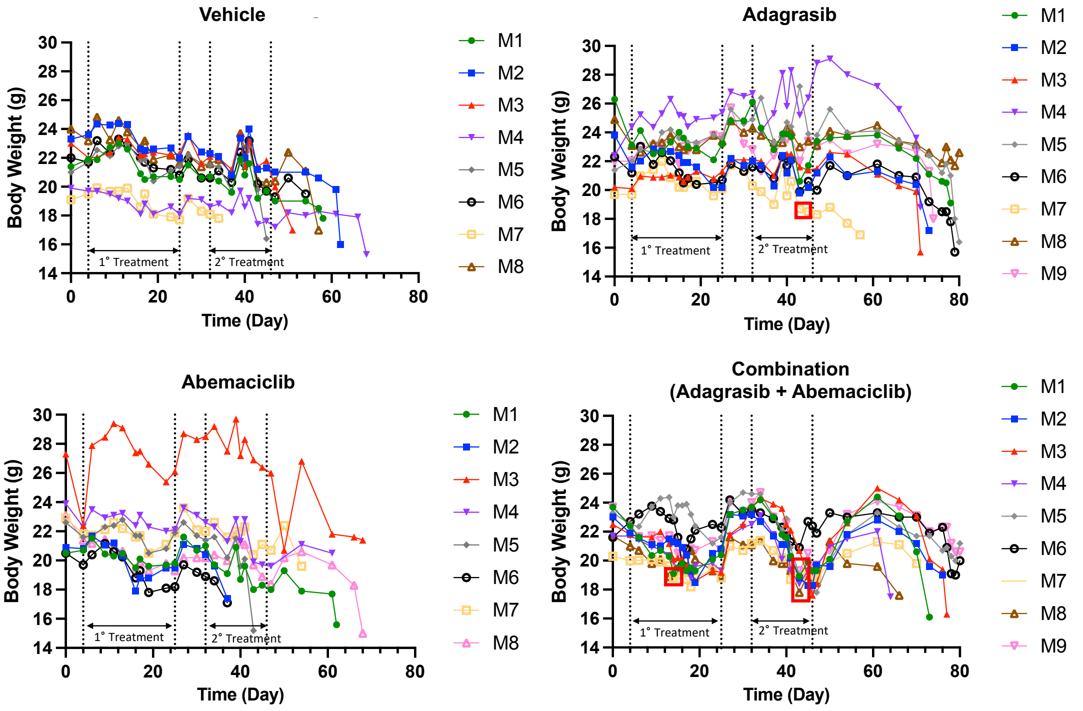

Supplementary Fig. 5. Body Weight Data (related with Figure 4). Mouse health monitoring with body weight measurements over the course of treatments. Mice were euthanized according to the criteria such as a 20% body weight loss, neurological signs, or a 15% body weight loss plus other signs. Adagrasib evening dosing was skipped in mice with impaired health conditions for the time period (days) shown by the red boxes.
